# Hemodynamic and Clinical Outcomes in Redo-Surgical Aortic Valve Replacement vs. Transcatheter Valve-in-Valve

**DOI:** 10.1016/j.shj.2022.100106

**Published:** 2022-10-28

**Authors:** Sébastien Hecht, Anne-Sophie Zenses, Jérémy Bernard, Lionel Tastet, Nancy Côté, Leonardo de Freitas Campos Guimarães, Jean-Michel Paradis, Jonathan Beaudoin, Kim O’Connor, Mathieu Bernier, Eric Dumont, Dimitri Kalavrouziotis, Robert Delarochellière, Siamak Mohammadi, Marie-Annick Clavel, Josep Rodés-Cabau, Erwan Salaun, Philippe Pibarot

**Affiliations:** Institut universitaire de cardiologie et de pneumologie de Québec/Québec Heart & Lung Institute, Universite Laval / Laval University, Québec City, Québec, Canada

**Keywords:** Aortic bioprosthesis, Redo-surgery, Structural valve deterioration, Transcatheter valve-in-valve

## Abstract

**Background:**

Transcatheter valve-in-valve replacement (ViV-TAVR) has emerged as an alternative to redo-surgical aortic valve replacement (Redo-SAVR) for the treatment of failed surgical aortic bioprostheses. However, the benefit of ViV-TAVR compared with Redo-SAVR remains debated with regard to short-term hemodynamic results and short- and long-term clinical outcomes.

**Objective:**

This study aimed to compare short-term hemodynamic performance and long-term clinical outcomes of ViV-TAVR vs. Redo-SAVR in patients treated for surgical aortic bioprosthetic valve failure.

**Methods:**

We retrospectively analyzed the data prospectively collected in 184 patients who underwent Redo-SAVR or ViV-TAVR. Transthoracic echocardiography was performed before and after the procedure and analyzed in an echocardiography core laboratory using the new Valve Academic Research Consortium-3 criteria. An inverse probability of treatment weighting was used to compare the outcomes between both procedures.

**Results:**

ViV-TAVR showed lower rate of intended hemodynamic performance (39.2% vs. 67.7%, *p* < 0.001) at 30 days, which was essentially driven by a higher rate (56.2% vs. 28.8%, *p* = 0.001) of high residual gradient (mean transvalvular gradient ≥20 mm Hg). Despite a trend for higher 30-day mortality in the Redo-SAVR vs. ViV-TAVR group (8.7% vs. 2.5%, odds ratio [95% CI]: 3.70 [0.77-17.6]; *p* = 0.10), the long-term mortality was significantly lower (24.2% vs. 50.1% at 8 years; hazard ratio [95% CI]: 0.48 [0.26-0.91]; *p* = 0.03) in the Redo-SAVR group. After inverse probability of treatment weighting analysis, Redo-SAVR remained significantly associated with reduced long-term mortality compared with ViV-TAVR (hazard ratio [95% CI]: 0.32 [0.22-0.46]; *p* < 0.001).

**Conclusions:**

ViV-TAVR was associated with a lower rate of intended hemodynamic performance and numerically lower mortality at 30 days but higher rates of long-term mortality compared with Redo-SAVR.

## Introduction

Bioprosthetic valves are the most frequently used valve substitutes for surgical aortic valve replacement (SAVR). However, their long-term durability is limited by structural valve deterioration.^1^ About one-third of bioprosthetic valves present signs of structural and hemodynamic deterioration at 10 years following SAVR, and 40% of the patients who survive up to 20 years after SAVR require an aortic valve reintervention for failed bioprosthetic valve.^1^, ^2^, ^3^

Redo-SAVR remains the standard of care for the treatment of failed surgical bioprosthetic valves but is associated with a higher risk of periprocedural complications and in-hospital mortality compared with the first SAVR. In recent years, transcatheter aortic valve-in-valve implantation (ViV-TAVR) has become a valuable option for the treatment of failed surgical aortic bioprostheses (BPs) in patients with prohibitive or high risk for Redo-SAVR.^4^, ^5^, ^6^, ^7^ However, there are no randomized studies that have compared the prosthetic valve hemodynamic performance post-reintervention and the long-term clinical outcomes between patients who underwent ViV-TAVR vs. Redo-SAVR. Some reports suggest higher rates of high residual transvalvular gradients and severe prosthesis-patient mismatch following the procedure, but probable similar or better short-term clinical outcomes with ViV-TAVR vs. Redo-SAVR.^8^^,^^9^

The objectives of this study were to compare hemodynamics using the new Valve Academic Research Consortium-3 (VARC-3) criteria and clinical outcomes of ViV-TAVR vs. Redo-SAVR at 30 days, at long-term follow-up, and to determine the factors associated with outcomes.

## Methods

### Study Population

In this observational single-center study, we retrospectively analyzed the data prospectively collected from 184 consecutive patients who underwent Redo-SAVR or ViV-TAVR for failed aortic surgical bioprosthetic valve due to structural valve deterioration between 2009 and 2017 at the Institut Universitaire de Cardiologie et de Pneumologie de Québec. The period of inclusion was chosen so that the last patient included in the study theoretically had at least 3 years of follow-up. Patients with reintervention for valve thrombosis, endocarditis, or paravalvular regurgitation were excluded. Additional concomitant coronary revascularization (coronary artery bypass graft [CABG]), ascending aorta intervention, and mitral or tricuspid valve repair/replacement were not considered as exclusion criteria.

Pre-reintervention, reintervention, and post-reintervention clinical data were prospectively gathered in an institutional database. However, the present study was not a prespecified analysis at the time of the setup of the database, and this study should thus be considered retrospective in nature. Transthoracic echocardiograms obtained prior to and 1 to 3 months after reintervention were retrospectively analyzed in an echocardiography core laboratory according to the VARC-3 criteria.^10^ The study was conducted in accordance with the Declaration of Helsinki, approved by the institutional review board, and because of its retrospective nature, informed written consent was not required.

### Echocardiographic Data

All echocardiographic measurements were performed using the TOMTEC Imaging platform (V.4.6, Image Arena TM, Munich, Germany) software. The mean transvalvular pressure gradient (MG), aortic valve area, and Doppler velocity index were measured with the Bernoulli equation and as recommended by guidelines.^11^ The severity grades of aortic (AR), mitral, and tricuspid valve regurgitations were assessed using a multiparameter integrative approach as previously described.^11^^,^^12^ The stroke volume was calculated by multiplying the cross-sectional area of the left ventricular outflow tract with the velocity-time integral measured below the prosthesis stent.^11^ The effective orifice area (EOA) was calculated using the continuity equation and indexed to body surface area. The left ventricular ejection fraction was measured using the biplane Simpson method.

Types of bioprosthetic valve dysfunction before reintervention were categorized as follows: (1) stenosis, if the mean gradient was ≥30 mm Hg without significant AR (≥moderate); (2) regurgitation, if AR was greater than or equal to moderate without significant stenosis (gradient <30 mm Hg); or (3) mixed, if mean gradient was ≥30 mm Hg with greater than or equal to moderate AR.

Because transthoracic echocardiogram images were not available at the time of the first SAVR, pre-existing prosthesis-patient mismatch (PPM) after the first SAVR was assessed using the predicted indexed EOA (i.e., the normal reference value of EOA for the model and size of BP implanted in the patient divided by the patient’s body surface area) as previously described.^11^ PPM post-reintervention was assessed using the EOAi measured at predischarge echocardiogram.^13^, ^14^, ^15^ PPM (preintervention and postintervention) was defined as not clinically significant (i.e., mild or no PPM) if the indexed EOA was >0.85 cm^2^/m^2^ (or >0.70 cm^2^/m^2^ if patients are obese: body mass index ≥30 kg/m^2^), moderate if it was >0.65 cm^2^/m^2^ but ≤0.85 cm^2^/m^2^ (or >0.55 cm^2^/m^2^ but ≤0.70 cm^2^/m^2^, if obese), and severe if it was ≤0.65 cm^2^/m^2^ (or ≤0.55 cm^2^/m^2^ if obese).

Intended hemodynamic performance (IHP) of the valve was defined by mean gradient <20 mm Hg, peak velocity <3 m/s, Doppler velocity index ≥0.25, and less than moderate AR measured at predischarge echocardiogram.^10^ Hemodynamic futility of reintervention was defined as a reduction in MG <10 mm Hg and no improvement in AR at predischarge echocardiogram vs. pre-reintervention echocardiogram.

### Study Endpoints

The primary study endpoints were (1) device success as defined by VARC-3^10^: composite of technical success, freedom from mortality at 30 days, freedom from surgery or intervention related to the device at 30 days, and IHP; and (2) all-cause mortality.

Mortality data were retrospectively obtained from the Québec Institute of Statistics. To maximize the interrogation of the central Québec Institute of Statistics database, a list with multiple demographics (including first and last names, date of birth, and social security number) and a delay of 1 year between interrogation and closing follow-up dates were used.

The secondary endpoints were (1) 30-day mortality, (2) IHP of the valve, (3) hemodynamic futility of reintervention, and (4) severe PPM post-reintervention.

### Statistical Analyses

Continuous variables were tested for normality by the Shapiro-Wilk or the Kolmogorov-Smirnov tests and expressed as mean ± standard deviation (or as median and interquartile range if not normally distributed). For normal and nonparametric continuous distributions, a Student's *t*-test and a Mann-Whitney test were performed, respectively. Categorical variables were compared using the chi-squared or Fisher's exact tests, as appropriate, and are expressed in number of patients with percentages. Univariable and multivariable regression models were used to identify factors associated with (1) device success at 30 days, (2) IHP post-reintervention, (3) severe PPM post-reintervention, and (4) all-cause mortality. Multivariable logistic regression analyses were first performed to determine the factors associated with device success, IHP, severe PPM in the global population, and then respectively in the ViV-TAVR and Redo-SAVR population. Survival curves were constructed using time-to-event Kaplan-Meier curves, and a log-rank test was also conducted for comparison between groups. Univariable Cox proportional hazards models were used to identify the factors associated with all-cause mortality following reintervention, and the results are presented as hazard ratio (HR) with 95% CIs. Comprehensive multivariable Cox analyses were limited in the number of risk factors that could be included in a single model because of the sample size and small number of deaths from any cause during the follow-up. We thus built 4 different models of interest to test the independent association of ViV-TAVR vs. Redo-SAVR with the primary endpoint. The first model includes clinical baseline variables most strongly associated with univariate analysis (i.e., age, sex, chronic obstructive pulmonary disease, renal insufficiency, and EuroSCORE II). The second model includes the EuroSCORE II and pre-reintervention echocardiographic known relevant variables or those with univariable *p* value <0.10. The third model includes the EuroSCORE II and post-reintervention echocardiographic relevant variables or those with *p* value <0.10, which can impact the long-term outcome (i.e., stroke volume index post-reintervention ≤35 mL/m^2^, mitral regurgitation or tricuspid regurgitation greater than or equal to moderate, and IHP). The fourth model includes the same variables as the third model but with device success instead of IHP. A propensity score was calculated from logistic regression, including age, sex, EuroSCORE II, and each baseline characteristic that differs between the treated populations (*p* value <0.20; Supplemental Table 1). The inverse probability of treatment weighting (IPTW) technique was computed for each patient from the propensity score previously calculated. Then, a multivariable Cox model was performed to confirm the factors independently associated with all-cause mortality following Redo-SAVR and ViV-TAVR. All statistical analyses were performed using SPSS 26.0 (IBM Corporation, Armonk, New York, New York), and a *p* value <0.05 was considered as statistically significant.

## Results

### Baseline Clinical and Echocardiographic Characteristics

Among the 184 patients who underwent reintervention for failed surgical aortic BPs, 104 (56.5%) underwent Redo-SAVR and 80 (43.5%) ViV-TAVR. Baseline characteristics of the study population according to the type of intervention are presented in Table 1.

**Table 1:** Baseline clinical characteristics of the total cohort and according to the type of reintervention

	Total cohort (N = 184)	Redo-SAVR (n = 104, 56.5%)	ViV-TAVR (n = 80, 43.5%)	*p* value
Age, years	72.3 ± 9.1	70.3 ± 7.3	74.4 ± 10.9	0.005
Age at first AVR, years	62.0 ± 9.76	61.0 ± 7.8	63.2 ± 11.7	0.118
Female sex, n (%)	65 (35.3)	38 (36.5)	27 (33.8)	0.695
Body surface area, m^2^	1.85 ± 0.22	1.87 ± 0.22	1.84 ± 0.22	0.362
Body mass index, kg/m^2^	28.3 ± 5.1	29.1 ± 5.1	27.6 ± 5.1	0.049
Hypertension, n (%)	152 (82.6)	85 (81.7)	67 (83.8)	0.720
Diabetes mellitus, n (%)	63 (34.2)	35 (33.7)	28 (35.0)	0.849
Previous myocardial infarction, n (%)	36 (19.6)	23 (22.1)	13 (16.3)	0.320
COPD, n (%)	35 (19.0)	15 (14.4)	20 (25.0)	0.070
Pacemaker, n (%)	20 (10.9)	2 (1.9)	18 (22.5)	<0.001
History of CABG, n (%)	73 (39.7)	32 (30.8)	41 (51.2)	0.005
Coronary artery disease, n (%)	100 (54.3)	45 (43.3)	55 (68.8)	<0.001
History of atrial fibrillation, n (%)	49 (26.6)	30 (28.8)	19 (23.8)	0.438
Cerebrovascular disease, n (%)	27 (14.7)	14 (13.5)	13 (16.3)	0.596
Renal insufficiency, n (%)	15 (8.2)	6 (5.8)	9 (11.3)	0.178
Creatinine Clearance, mL/min/1.73m^2^	66 ± 21	68 ± 20	65 ± 22	0.120
Creatinine level				0.262
0-120 mg/dL	139 (75.5)	83 (79.8)	56 (70.0)	-
121-180 mg/dL	38 (20.7)	17 (16.3)	21 (26.3)	-
>180 mg/dL	7 (3.8)	4 (3.8)	3 (3.8)	-
EuroSCORE II	8.6 (4.6-15.3)	9.3 (4.8-18.8)	7.5 (3.8-13.0)	0.471
NYHA functional class, n (%)				0.884
I	11 (6.0)	7 (6.7)	4 (5.0)	-
II	51 (27.7)	29 (27.9)	22 (27.5)	-
III	101 (54.9)	55 (52.9)	46 (57.5)	-
IV	21 (11.4)	13 (12.5)	8 (10.0)	-

*Notes*. Continuous data are expressed by mean ± SD or median (25th to 75th interquartile range). Categorical data are expressed by number (percentage). *p* values refer to comparison between Redo-SAVR and ViV-TAVR.

AVR, aortic valve replacement; CABG, coronary artery bypass grafting; COPD, chronic obstructive pulmonary disease; EuroSCORE II, European System for Cardiac Operative Risk Evaluation II; ​NYHA, New York Heart Association functional class; Redo-SAVR, redo-surgical aortic valve replacement; ViV-TAVR, valve-in-valve transcatheter aortic valve replacement.

The mean age of the total cohort was 72.3 ± 9.1 years, and 64.7% were men. Patients who underwent ViV-TAVR were older (74.4 ± 10.9 vs. 70.3 ± 7.3 years; *p* = 0.005), had more pacemaker pre-reintervention (22.5% vs. 1.9%, *p* < 0.001), and prior CABG (51.2% vs. 30.8%, *p* = 0.005) compared with those who underwent Redo-SAVR (Table 1). Other clinical baseline characteristics were comparable between groups (Table 1).

Baseline echocardiographic data are presented in Table 2. The median time from the first SAVR to reintervention was 8.9 (5.8-12.9) years in the Redo-SAVR group compared with 10.8 (7.4-14.9) in the ViV-TAVR group (*p* < 0.001; Supplemental Table 2). However, echocardiographic data at the time of reintervention were not significantly different between groups (Table 2). The proportion of stented vs. stentless surgical BPs was similar between groups, but patients who underwent ViV-TAVR had more frequently a surgical BP size ≤21 mm (32.5% vs. 12.5%, *p* = 0.001) and pre-existing moderate or severe PPM (39.1% vs. 22.1%, *p* = 0.02; Supplemental Table 2).

**Table 2:** Baseline echocardiographic characteristics of the total cohort and according to the type of reintervention

	Total cohort (N = 184)	Redo-SAVR (n = 104, 56.5%)	ViV-TAVR (n = 80, 43.5%)	*p* value
Time to bioprosthesis failure, years	10.2 (6.7-13.7)	8.9 (5.8-12.9)	10.8 (7.4-14.9)	<0.001
Surgical bioprosthesis size (≤21 mm), n (%)	39 (21.2)	13 (12.5)	26 (32.5)	0.001
Internal orifice diameter, mm	20.7 ± 2.67	21.1 ± 2.87	19.9 ± 2.12	0.005
Pre-existing PPM, n (%)				0.045
None	123 (71.1)	81 (77.9)	42 (60.9)	-
Moderate	48 (27.7)	22 (21.1)	26 (37.6)	-
Severe	2 (1.2)	1 (1.0)	1 (1.5)	1.000
Hemodynamic valve deterioration type, n (%)				0.738
Stenosis	80 (43.5)	47 (45.2)	33 (41.2)	-
Regurgitation	65 (35.3)	37 (35.6)	28 (35.0)	-
Mixed	39 (19.6)	20 (23.8)	19 (21.2)	-
Aortic transvalvular MG, mm Hg	32.0 (19.1-48.8)	31.7 (19.1-48.7)	34.0 (19.3-48.9)	0.642
Aortic transvalvular MG ≥ 20 mm Hg, n (%)	133 (74.7)	73 (74.5)	60 (75.0)	0.942
Doppler velocity index	0.24 (0.19-0.35)	0.25 (0.19-0.36)	0.24 (0.18-0.35)	0.602
Stroke volume index, mL/m^2^	41.2 (36.4-49.8)	40.1 (35.1-49.2)	41.8 (37.8-50.6)	0.193
EOA, cm^2^	1.07 ± 0.53	1.09 ± 0.55	1.04 ± 0.51	0.551
EOAi, cm^2^/m^2^	0.57 ± 0.29	0.57 ± 0.28	0.57 ± 0.29	0.962
Aortic regurgitation, n (%)				0.168
None	18 (9.8)	11 (10.6)	7 (8.8)	-
Trace	36 (19.6)	21 (20.2)	15 (18.8)	-
Mild	24 (13.0)	13 (12.5)	11 (13.8)	-
Moderate	43 (23.4)	17 (16.3)	26 (32.5)	-
Severe	58 (31.5)	37 (35.6)	21 (26.2)	-
Left ventricular ejection fraction, %	61.0 (51.1-66.2)	61.6 (50.0-65.9)	61.0 (52.1-67.1)	0.731
Mitral regurgitation, n (%)				0.930
None	3 (1.8)	1 (1.0)	2 (2.5)	-
Trace	54 (29.3)	30 (34.5)	24 (30.4)	-
Mild	78 (42.4)	41 (47.1)	37 (46.8)	-
Moderate	27 (14.7)	13 (14.9)	14 (17.7)	-
Severe	4 (2.4)	2 (2.3)	2 (2.5)	-
Tricuspid regurgitation ≥ moderate, n (%)	23 (13.9)	10 (11.5)	13 (16.7)	0.338
Systolic pulmonary artery pressures ≥50 mm Hg, n (%)	40 (33.6)	19 (30.6)	21 (36.8)	0.470
Annular tricuspid systolic wave, cm/s	10.0 ± 2.3	10.2 ± 2.2	9.8 ± 2.4	0.357

EOA, effective orifice area; MG, mean transvalvular pressure gradient; PPM, prosthesis-patient mismatch.

### Procedural and In-Hospital Data

One-third of the patients underwent concomitant coronary revascularization (CABG or PCI) during reintervention, without a difference between groups (Supplemental Table 3). Seventy-five (72.1%) patients underwent a concomitant procedure on the aorta or the other valves during the Redo-SAVR, including aortic root enlargement or replacement in 22 (21.1%) patients, ascending aorta replacement in 25 (24.0%) patients, mitral valve repair or replacement in 22 (21.1%), and tricuspid valve repair in 6 (5.8%) (Supplemental Table 3). In patients who underwent ViV-TAVR, transfemoral access was used in 47 (58.8%) patients, transapical in 24 (30.0%), and other in 9 (11.2%); 50 (62.5%) received a SAPIEN transcatheter heart valve (THV), 28 (35.0%) a CoreValve THV, and 2 (2.5%) a Portico THV (Supplemental Table 3). Fracturing of the surgical bioprosthetic valve stent at the time of ViV-TAVR was not performed in any of the patients included in this series.

Procedural and in-hospital complications are presented in Supplemental Table 4. The new onset of atrial fibrillation was more frequent in patients who underwent Redo-SAVR compared with those with ViV-TAVR (29 [28.2%] vs. 5 [6.8%], *p* < 0.001). The hospital stay duration (from admission to discharge) was longer for patients with Redo-SAVR vs. those with ViV-TAVR (9.7 ± 5.5 days vs. 6.8 ± 4.9 days; *p* < 0.001).

### Device Success and Intended Valve Hemodynamic Performance

Post-reintervention, patients who underwent ViV-TAVR had higher aortic MG (22.1 ± 9.3 mm Hg vs. 18.2 ± 10.4 mm Hg, *p* = 0.01), smaller valve EOA (1.11 ± 0.39 vs. 1.29 ± 0.41 cm^2^, *p* = 0.004), but similar rate of total AR greater than or equal to moderate (3 [3.8%] vs. 1 [1.0%], *p* = 0.20) in comparison with Redo-SAVR patients (Table 3). Following ViV-TAVR procedure, the rate of high residual gradient was not significantly different according to the type of THV (balloon expandable [59.2%] and self-expanding [53.3%], *p* = 0.61). There was a trend for higher rate of high residual gradient with small transcatheter valve size (i.e., ≤23 ​mm) vs. larger transcatheter valve sizes (64.2% vs. 42.3%, *p* = 0.06, respectively). IHP of the valve was achieved in 39.2% of ViV-TAVR vs. 67.7% of Redo-SAVR, *p* < 0.001. Severe PPM following reintervention was more frequent in ViV-TAVR vs. Redo-SAVR (47 [59.5%] vs. 35 [41.7%], *p* < 0.001), whereas the rate of hemodynamic futility of reintervention (7.8% vs. 7.0%, *p* = 0.84) was not significantly different between groups (Supplemental Figure 1). Moreover, the rate of moderate or severe mitral regurgitation postprocedure was higher in patients with ViV-TAVR compared with those with Redo-SAVR (9.1% vs. 2.2%, *p* = 0.046). The rate of device success was significantly lower in ViV-TAVR vs. Redo-SAVR (35.0% vs. 59.6%, *p* < 0.001).

**Table 3:** Postprocedural (1-3 months) echocardiographic results according to type of reintervention

	Total cohort (N = 184)	Redo-SAVR (n = 104, 56.5%)	ViV-TAVR (n = 80, 43.5%)	*p* value
Echocardiographic parameters				-
Aortic transvalvular MG, mm Hg	20.0 ± 10.1	18.2 ± 10.4	22.1 ± 9.3	0.012
Doppler velocity index	0.35 ± 0.21	0.40 ± 0.28	0.30 ± 0.08	0.004
Stroke volume index, mL/m^2^	36.6 ± 8.9	35.0 ± 8.6	38.3 ± 8.9	0.013
EOA, cm^2^	1.20 ± 0.40	1.29 ± 0.41	1.11 ± 0.39	0.004
EOAi, cm^2^/m^2^	0.65 ± 0.22	0.69 ± 0.22	0.61 ± 0.22	0.002
Left ventricular ejection fraction, %	61.0 (50.7-65.8)	61.4 (51.0-65.3)	60.3 (50.2-66.9)	0.689
Mitral regurgitation, n (%)				0.239
None	7 (4.1)	4 (4.3)	3 (3.9)	-
Trace	90 (53.3)	48 (52.2)	42 (54.5)	-
Mild	63 (37.3)	38 (41.3)	25 (32.5)	-
Moderate	7 (4.1)	1 (1.1)	6 (7.8)	-
Severe	2 (1.2)	1 (1.1)	1 (1.3)	-
Mitral regurgitation ≥ moderate, n (%)	9 (5.3)	2 (2.2)	7 (9.1)	0.046
Tricuspid regurgitation ≥ moderate, n (%)	28 (15.2)	11 (10.6)	17 (21.3)	0.092
Systolic pulmonary artery pressures ≥50 mm Hg, n (%)	23 (17.3)	9 (12.9)	14 (22.2)	0.154
Annular tricuspid S wave, cm/s	9.3 ± 2.3	8.9 ± 2.1	9.8 ± 2.6	0.060
Changes from pre-reintervention				-
ΔMG, mm Hg	−11.9 (−30.1 to −2.0)	−15.7 (−32.8 to −3.8)	−9.35 (−23.0 to −0.1)	0.081
ΔEOA, cm^2^	+0.18 (−0.11 to 0.45)	+0.26 (−0.06 to 0.58)	+0.11 (−0.21 to 0.33)	0.048
ΔEOAi, cm^2^/m^2^	+0.10 (−0.09 to 0.23)	+0.14 (−0.04 to 0.30)	+0.05 (−0.12 to 0.19)	0.050
ΔStroke volume index, mL/m^2^	−6.1 (−13.6 to −0.5)	−7.9 (−15.7 to 0.3)	−4.0 (−12.3 to −1.0)	0.282
ΔLeft ventricular ejection fraction, %	−1.20 (−5.95 to 2.96)	−0.99 (−7.99 to 4.57)	−1.77 (−4.68 to 2.47)	0.854
Aortic valve hemodynamic function				-
Device success, n (%)	90 (48.9)	62 (59.6)	28 (35.0)	<0.001
Intended hemodynamic performance, n (%)	94 (54.7)	63 (67.7)	31 (39.2)	<0.001
Aortic transvalvular MG ≥ 20 mm Hg, n (%)	75 (40.8)	30 (28.8)	45 (56.2)	0.001
Total aortic regurgitation ≥ moderate, n (%)	4 (2.2)	1 (1.0)	3 (3.8)	0.200
PPM, n (%)				0.072
None	37 (22.7)	23 (27.4)	14 (17.7)	-
Moderate	44 (27.0)	26 (31.0)	18 (22.8)	-
Severe	82 (50.3)	35 (41.7)	47 (59.5)	<0.001
Hemodynamic futility, n (%)	12 (7.4)	6 (7.0)	6 (7.8)	0.840

EOA, effective orifice area; MG, mean transvalvular pressure gradient; PPM, prosthesis-patient mismatch; Redo-SAVR, redo-surgical aortic valve replacement; ViV-TAVR, valve-in-valve transcatheter aortic valve replacement.

In the overall study population (ViV-TAVR + Redo-SAVR), the factors associated with device success, IHP, and severe PPM following reintervention in univariate analysis are presented in Table 4. In multivariable analysis, Redo-SAVR (vs. ViV-TAVR) and BP mode of failure (regurgitation vs. stenosis or mixed) were the only factors independently associated with higher rate of device success (odds ratio [OR; 95% CI]: 2.38 [1.18-4.76], *p* = 0.016 and OR [95% CI]: 2.63 [1.25-5.55], *p* = 0.010, respectively). Redo-SAVR (vs. ViV-TAVR) was also independently associated with higher rate of IHP (OR [95% CI]: 3.25 [1.74-6.09], *p* = 0.003); and pre-existing PPM (i.e., PPM of the surgical BP) greater than or equal to moderate was the only factor independently associated with severe PPM postreintervention (OR [95% CI]: 3.11 [1.37-7.08], *p* = 0.007; Table 4).

**Table 4:** Factors associated with device success, intended hemodynamic valve performance, and severe prosthesis-patient mismatch in the total cohort

	Univariate OR (95% CI)	*p* value	Multivariate OR (95% CI)	*p* value
Factors associated with device success at 30-d				-
ViV-TAVR vs. Redo-SAVR	0.36 (0.20-0.67)	0.001	0.42 (0.21-0.85)	0.016
EuroSCORE II	1.00 (0.98-1.03)	0.704	0.99 (0.96-1.02)	0.376
Hypertension	0.51 (0.23-1.12)	0.094	0.70 (0.27-1.83)	0.469
History of atrial fibrillation	1.76 (0.91-3.41)	0.095	1.53 (0.70-3.33)	0.285
BP mode of failure (AS or mixed vs. AR)	0.41 (0.22-0.76)	0.005	0.38 (0.18-0.80)	0.010
Pre-existing PPM ≥ moderate	0.44 (0.22-0.87)	0.018	0.55 (0.26-1.16)	0.119
Factors associated with intended hemodynamic valve performance				-
ViV-TAVR vs. Redo-SAVR	0.31 (0.16-0.58)	<0.001	0.32 (0.15-0.68)	0.003
Age	0.99 (0.95-1.02)	0.513	1.01 (0.97-1.05)	0.674
Female sex	0.83 (0.44-1.56)	0.565	0.78 (0.38-1.59)	0.492
Hypertension	0.40 (0.17-0.93)	0.034	0.58 (0.22-1.52)	0.265
History of atrial fibrillation	2.17 (1.07-4.39)	0.032	1.55 (0.71-3.40)	0.270
BP mode of failure (AS or mixed vs. AR)	0.46 (0.24-0.89)	0.022	0.51 (0.24-1.07)	0.076
Pre-existing PPM ≥ moderate	0.41 (0.20-0.82)	0.012	0.54 (0.26-1.15)	0.110
Factors associated with severe prosthesis-patient mismatch				-
ViV-TAVR vs. Redo-SAVR	2.18 (1.17-4.06)	0.008	1.77 (0.81-3.90)	0.155
Age	1.04 (1.00-1.07)	0.035	1.01 (0.97-1.06)	0.517
Female sex	1.28 (0.67-2.43)	0.445	1.31 (0.62-2.77)	0.480
Hypertension	1.88 (0.83-4.26)	0.127	1.10 (0.42-2.87)	0.843
History of atrial fibrillation	0.43 (0.22-0.88)	0.021	0.45 (0.20-1.03)	0.058
Clinical Pacemaker	3.65 (1.26-10.57)	0.017	2.81 (0.71-11.02)	0.139
COPD	0.55 (0.25-1.21)	0.137	0.45 (0.17-1.20)	0.111
Pre-existing PPM ≥ moderate	4.22 (1.96-9.05)	<0.001	3.11 (1.37-7.08)	0.007

AR, aortic regurgitation; AS, aortic stenosis; BP, bioprosthesis; COPD, chronic obstructive pulmonary disease; EuroSCORE II, European System for Cardiac Operative Risk Evaluation II; OR, odds ratio; PPM, prosthesis-patient mismatch; Redo-SAVR, redo-surgical aortic valve replacement; ViV-TAVR, valve-in-valve transcatheter aortic valve replacement.

In an analysis restricted to the ViV-TAVR group, the mode of failure of the surgical BP (stenosis or mixed vs. regurgitation) was independently associated with lower rate of device success (OR [95% CI]: 0.28 [0.87-0.90], *p* = 0.032) and IHP (OR [95% CI]: 0.28 [0.09-0.87], *p* = 0.028) following reintervention (Supplemental Table 5). Pre-existing PPM greater than or equal to moderate was independently associated with severe PPM post-reintervention (OR [95% CI]: 7.58 [1.85–31.07], *p* = 0.005, Supplemental Table 5). In the Redo-SAVR group, none of the baseline factors were independently associated with device success, IHP, or severe PPM in multivariable analysis (Supplemental Table 6).

### Short- and Long-Term Clinical Outcomes

During a median follow-up of 5.6 years (interquartile range: 3.8-8.2 years), 61 patients died. There was a trend toward higher mortality rates at 30 days (8.7 vs. 2.5%; *p* = 0.08; OR [95% CI]: 3.70 [0.77-17.6], *p* = 0.10) and 1 year (12.5% vs. 7.5%; *p* = 0.27; OR [95% CI]:1.76 [0.64-4.86], *p* = 0.27) in Redo-SAVR vs. ViV-TAVR (Supplemental Table 4). IPTW analyses confirmed the absence of significant difference between Redo-SAVR vs. ViV-TAVR (30-day mortality: OR [95% CI]: 1.44 [0.59-3.52], *p* = 0.42; 1-year mortality: OR [95% CI]: 1.23 [0.64-2.36], *p* = 0.54).

**Figure 1:**
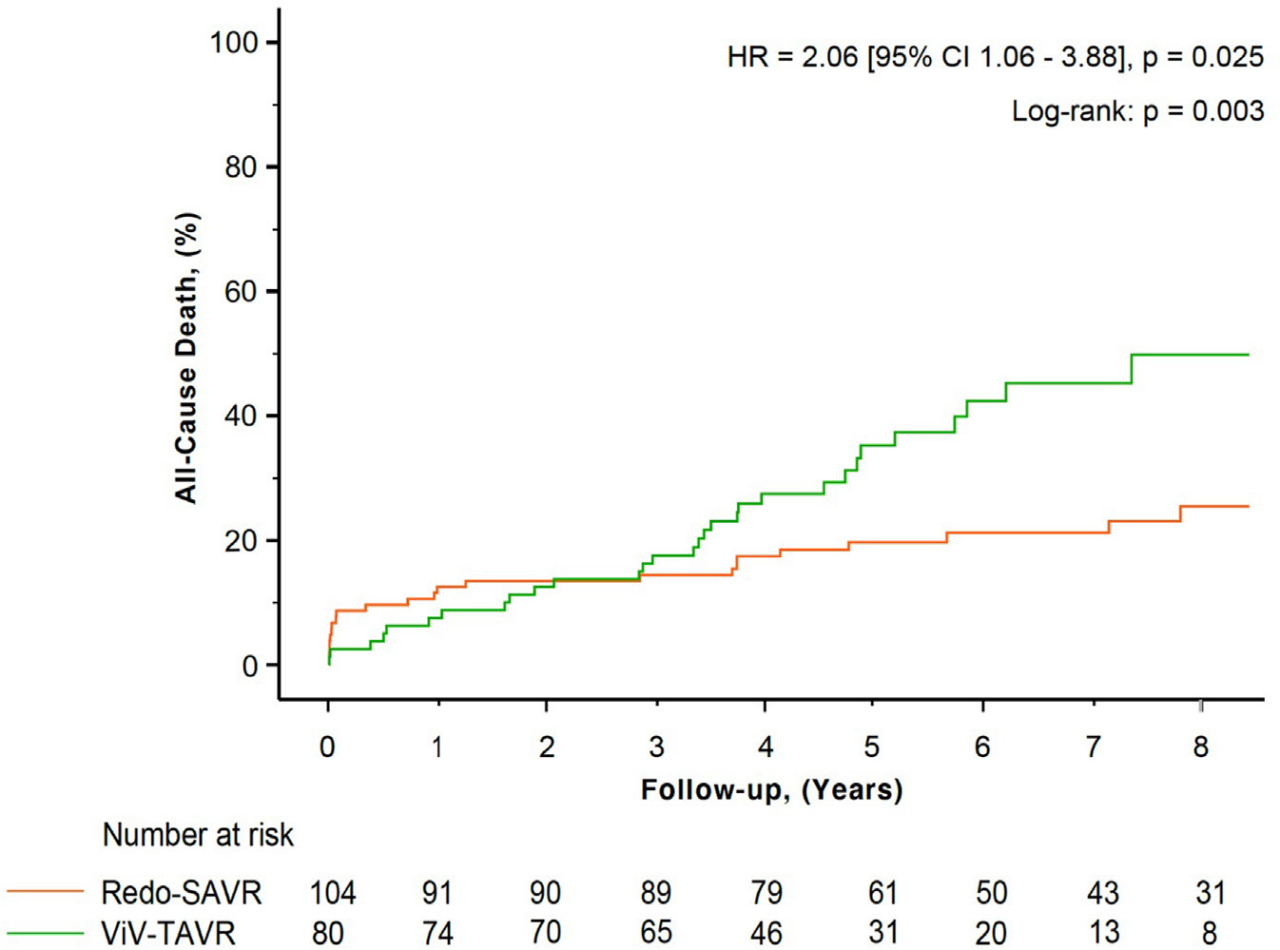
**All-cause mortality according to the type of reintervention**. Postprocedural all-cause death (%) following Redo-SAVR (orange line) and ViV-TAVR (green line). There was a significant difference between groups (log-rank *p* = 0.003) and a significantly higher rate of death in the ViV-TAVR group vs. Redo-SAVR (HR [95% CI]: 2.06 [1.06-3.88], *p* = 0.025). Abbreviations: HR, hazard ratio; Redo-SAVR, redo-surgical aortic valve replacement; ViV-TAVR, transcatheter valve-in-valve.

**Figure 2:**
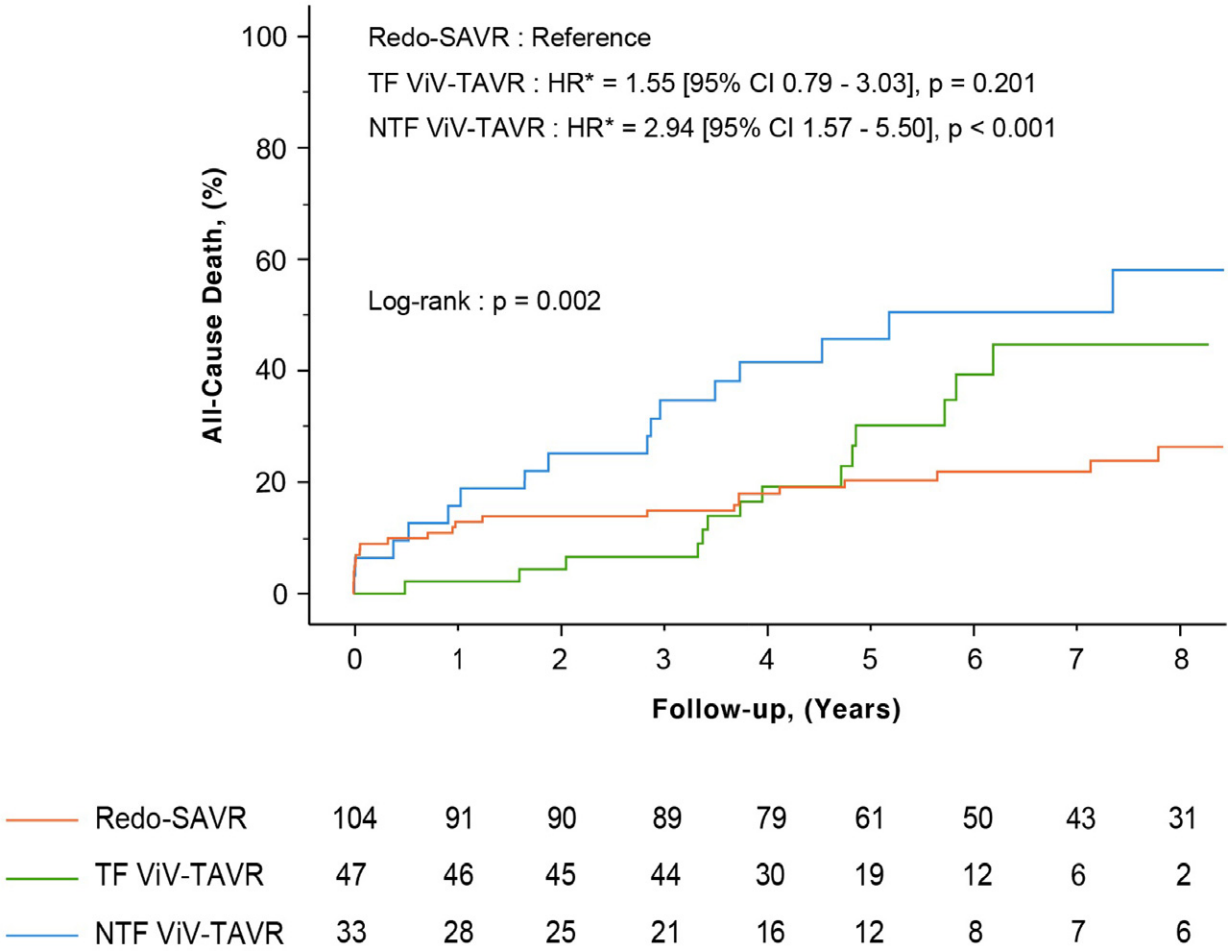
**Postprocedural all-cause death according to the type of reintervention and the arterial access**. Postprocedural all-cause death (%) following Redo-SAVR (orange line), TF ViV-TAVR (green line) and NTF ViV-TAVR (blue line). There was a significant difference between groups (log-rank *p* = 0.002). ∗Unadjusted HR. Abbreviations: NTF, nontransfemoral; Redo-SAVR, redo-surgical aortic valve replacement; TF, transfemoral; ViV-TAVR, transcatheter valve-in-valve.

**Figure 3:**
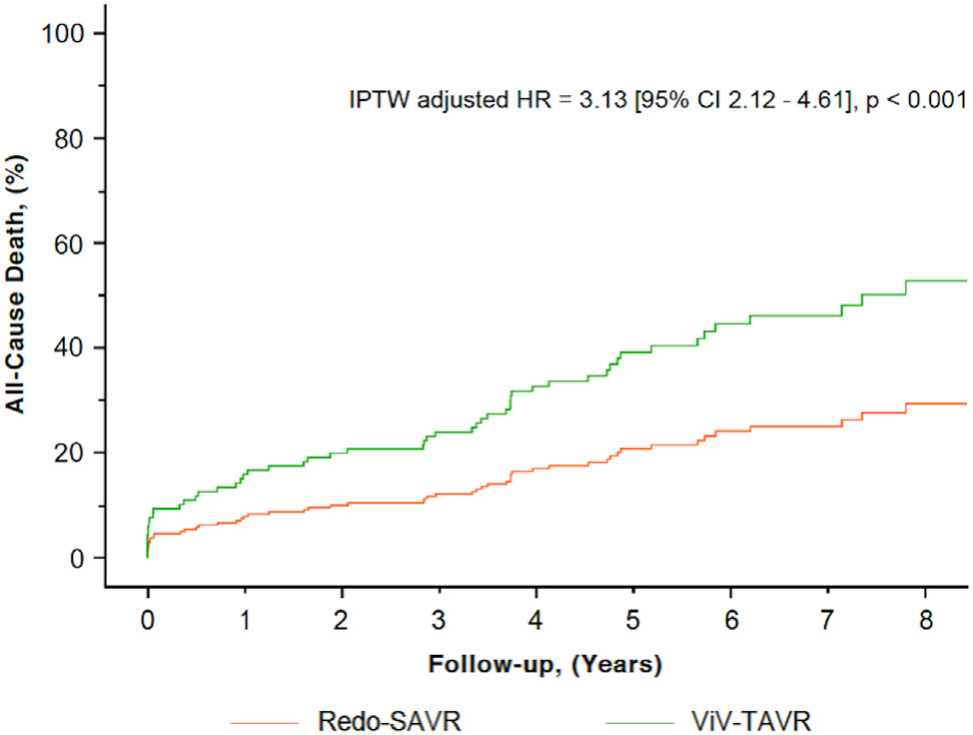
**IPTW-adjusted all-cause mortality curve according to the type of reintervention**. Inverse probability treatment weighting–adjusted Cox curve of all-cause mortality according to the type of reintervention (Redo-SAVR [orange line] and ViV-TAVR [green line]). There was a significantly higher rate of death in the ViV-TAVR group vs. Redo-SAVR (HR [95% CI]: 3.13 [2.12-4.61], *p* < 0.001). Abbreviations: HR, hazard ratio; IPTW, inverse probability treatment weighting; Redo-SAVR, redo-surgical aortic valve replacement; ViV-TAVR, transcatheter valve-in-valve.

During the long-term follow-up, ViV-TAVR (vs. Redo-SAVR) was independently associated with an increased risk of all-cause mortality (HR [95% CI]: 2.06 [1.06-3.88], *p* = 0.025; Figure 1). However, ViV-TAVR performed by transfemoral access had better survival compared with ViV-TAVR performed by nontransfemoral access (Figure 2). Redo-SAVR with a concomitant procedure on the mitral valve and/or tricuspid valve was associated with higher risk of short-term mortality compared with Redo-SAVR with any other surgical concomitant procedure (Supplemental Figure 2). The factors associated with all-cause mortality in univariable analyses are presented in Supplemental Table 7. In multivariable analyses, ViV-TAVR remained significantly associated with increased risk of long-term mortality in all tested models (Supplemental Table 8). With IPTW, ViV-TAVR (vs. Redo-SAVR) remained associated with higher risk of all-cause mortality (HR [95% CI]: 3.13 [2.12-4.61], *p* < 0.001, Figure 3). Subgroup analyses revealed no significant interaction between baseline factors and type of reintervention (ViV-TAVR vs. Redo-SAVR) with respect to impact on mortality (Supplemental Figure 3). The percentage of patients requiring reintervention was similar following Redo-SAVR (n = 5; 4.8%) vs. ViV-TAVR (n = 7; 8.8%). Older age (OR [95% CI]: 0.90 [0.85-0.96], *p* < 0.001) and previous history of CABG (OR [95% CI]: 0.13 [0.02-1.00], *p* = 0.050) were associated with lower risk of reintervention (Supplemental Table 9).

## Discussion

The main findings of this study are (1) ViV-TAVR is associated with a lower rate of device success, IHP, and higher rates of postprocedural severe PPM and high residual gradients compared with Redo-SAVR, whereas the rates of moderate/severe AR and hemodynamic futility of reintervention were low and not significantly different between groups; (2) There was a trend toward lower 30-day and 1-year mortality with ViV-TAVR vs. Redo-SAVR; (3) Redo-SAVR is independently associated with better long-term survival compared with ViV-TAVR, confirmed by IPTW-adjusted analyses.

### Early Hemodynamic Outcome of ViV-TAVR vs. Redo-SAVR

The present study confirms and expands previous reports suggesting that Redo-SAVR has greater potential to improve valve EOA and thus decrease gradients, compared with ViV-TAVR in patients with failed bioprosthetic valve.^8^^,^^9^^,^^16^ On the one hand, ViV-TAVR significantly improves aortic valve hemodynamics with a low and similar rate of hemodynamic futility compared with Redo-SAVR (7.8% vs. 7.0%). On the other hand, ViV-TAVR was associated with higher rates of severe PPM and high residual gradients, leading to superiority of Redo-SAVR over ViV-TAVR with regard to IHP (Redo-SAVR [67.7%] vs. ViV-TAVR [39.2%]; *p* < 0.001; OR [95% CI]: 3.25 [1.74-6.09]; *p* < 0.001) and device success (59.6% vs. 35.0%), despite a similar and low rate of moderate/severe AR in both groups.

The nature of both interventions, by definition, a new valve implanted within the failed surgical BP that is left in place in case of ViV-TAVR, compared with an explanation of the failed valve replaced by a new one in case of Redo-SAVR, explain the difference in hemodynamics post-reintervention and also the risk of worse hemodynamics when ViV-TAVR is performed in stenotic (or mixed) failed bioprosthetic valve (compared with regurgitant valves). In comparison to the study recently published by Landes et al,^17^ the present study reports a higher prevalence of high residual gradient (56.2% vs. 21.5%) and a smaller average EOA (1.11 cm^2^ vs. 1.37 cm^2^) in ViV-TAVR patients. These differences may be, at least in part, explained by the higher prevalence of patients who received a small (≤21 mm) surgical aortic valve BP at the time of the first aortic valve replacement (32.5% vs. 24.8%) and the much higher rate of use of balloon-expandable transcatheter valves for the ViV-TAVR (62.5% vs. 30.3%) in our study. To date, the clinical relevance and impact of severe PPM and high residual gradients following ViV-TAVR remain unclear,^14^^,^^18^, ^19^, ^20^ in the present study, there was no association between IHP, severe PPM, or high residual gradients at predischarge echocardiogram and 1-year or long-term outcomes. Furthermore, the overestimation of PPM and transprosthetic gradients by Doppler echocardiography, which appears to be more common following ViV-TAVR vs. de novo TAVR or SAVR,^14^^,^^20^ may play a role in these findings. Hence, both types of reinterventions achieve significant improvement in valve hemodynamics in the vast majority of patients with failed surgical BPs. Although device success was associated with better survival, this association did not remain statistically significant in multivariable analysis (Supplemental Tables 7 and 8).

### Short- and Long-Term Clinical Outcomes of ViV-TAVR vs. Redo-SAVR

The lower risk of pacemaker implantation, new onset of atrial fibrillation, renal failure, stroke/TIA, shorter hospital stays, and lower in-hospital and short-term mortality following ViV-TAVR vs. Redo-SAVR observed in the present study are consistent with the results of recent studies and meta-analyses.^4^, ^5^, ^6^, ^7^^,^^9^^,^^16^^,^^21^ These excellent short-term clinical outcomes of ViV-TAVR promote the adoption of this less invasive strategy for the treatment of failed bioprosthetic valves in patients with high surgical risk.^22^^,^^23^

**Figure 4:**
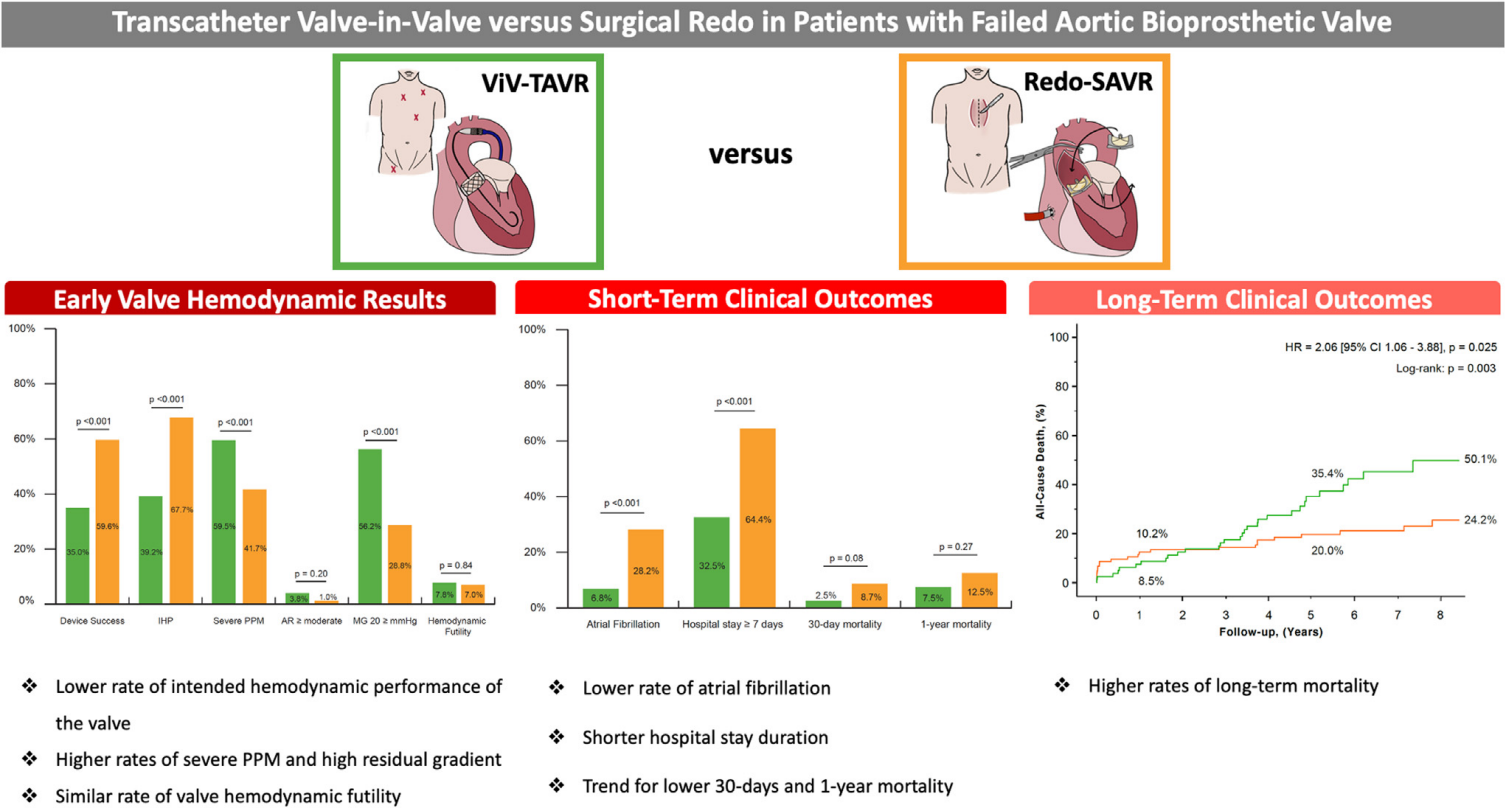
**Transcatheter valve-in-valve vs. surgical redo in patients with failed aortic bioprosthetic valve**. Hemodynamic and clinical outcomes following Redo-SAVR and ViV-TAVR. Abbreviations: AR, aortic regurgitation; HR, hazard ratio; IHP, intended hemodynamic performance; MG, mean transvalvular gradient; PPM, patient-prosthesis mismatch; Redo-SAVR, redo-surgical aortic valve replacement; ViV-TAVR, transcatheter valve-in-valve.

However, data at mid- and long-term are too conflicting to give a clear recommendation between ViV-TAVR and Redo-SAVR in patients at intermediate risk and low risk with longer expected life expectancy. Whereas Tam et al reported better 5-year survival rates in patients treated by ViV-TAVR vs. Redo-SAVR,^4^ Deharo et al^5^ found higher rates of heart failure, rehospitalization, and mortality beyond 1-year post-ViV-TAVR compared with Redo-SAVR. In the present study, a crossing of the mortality curves at approximately 3 years postprocedure was found, with lower mortality rates in ViV-TAVR vs. Redo-SAVR before 3 years and higher rates thereafter (Figure 4). Several factors may have contributed to this observation: first, nontransfemoral access was used in 41% of the ViV-TAVR patients included in the present series that was initiated in 2009 to have a long-term clinical follow-up. In randomized trials, transfemoral de novo TAVR is associated with similar or even better outcomes than SAVR, whereas transapical or other nontransfemoral TAVR access is known to be associated with worse outcomes, and these results are consistent with our findings.

Second, in the present investigation, as well as in several other previous studies,^24^^,^^25^ the early valve hemodynamic factors were not associated with subsequent clinical outcomes.

Third, despite a similar rate of concomitant coronary revascularization procedures in ViV-TAVR vs. Redo-SAVR, the effectiveness of revascularization by CABG (compared with PCI) may have contributed to reduce ischemic events and mortality in the long-term follow-up. Moreover, although a more aggressive surgical strategy of concomitant mitral and/or tricuspid valve intervention is associated with higher short-term mortality, the reduction of the rate of mitral or tricuspid regurgitations greater than or equal to moderate in patients with Redo-SAVR may have brought a long-term benefit. Hence, the absence or incompleteness of concomitant procedures to treat pre-existing comorbidities at the time of ViV-TAVR may be one of the main factors leading to the excess mortality observed beyond 3 years in this group.

### Clinical Implication and the Pivotal Role of the Heart Team

Based on the findings of the present study but also those previously reported, the role of the heart team and an individual case-by-case approach are strongly reinforced. The consideration of several factors by the heart team including surgical risk, pre-existing severe PPM, mode of surgical BP failure, type of bioprosthetic valve, comorbidities (such as complex coronary artery disease, and significant mitral or tricuspid valve regurgitation), and feasibility of transfemoral access; is key to selecting the most appropriate type of reintervention (Supplemental Figure 4).

ViV-TAVR should likely be considered in patients with (1) prohibitive or very high surgical risk; or (2) low, intermediate, or high surgical risk having none of the risk markers mentioned previously. To improve the long-term outcomes in patients referred for ViV-TAVR, concomitant or staged coronary and other concomitant valve procedures such as BP stent fracture should be considered in this population. Previous studies have reported that stent fracture improves hemodynamics and reduces the rate of severe PPM or high gradients.^26^^,^^27^

### Limitations

Although the present study provides a first overview of the long-term comparison between ViV-TAVR and Redo-SAVR for structural bioprosthetic failure, uses for the first time the new VARC-3 criteria in this field of research, and adds valuable information compared with previous studies, several limitations should be noted.

This is a retrospective and observational single-center study, which may have introduced biases including treatment allocation bias. Moreover, to obtain a long-term follow-up, this study was conducted over a large period of screening (2009-2017). The study was designed to reflect the real practice during the last decade, but the nonrandomized design may also have induced some biases regarding the evolution of clinical practice (several generations of THV, high prevalence of transapical implantation in the early experience, etc.). In the future, the increasing use of minimally invasive access such as transfemoral and carotid access may contribute to improve short- and long-term clinical outcomes of ViV-TAVR.

There were several differences in the baseline characteristics between the 2 treatment groups including longer from first aortic valve replacement to surgical BP failure and higher frequency of small surgical BPs and of pre-existing PPM. We attempted to adjust for these differences using IPTW, but we cannot exclude that some unmeasured confounding factors may be present and different between ViV-TAVR vs. Redo-SAVR.

## Conclusions

On the one hand, ViV-TAVR was associated with a lower rate of IHP, which was essentially driven by higher rate of high residual gradients compared with Redo-SAVR. On the other hand, the rates of moderate/severe AR and of hemodynamic futility of reintervention were low and not significantly different between groups. Although there was a trend for lower 30-day and 1-year mortality with ViV-TAVR vs. Redo-SAVR, the latter was associated with better long-term survival compared with ViV-TAVR. These results emphasized that the choice between ViV-TAVR and Redo-SAVR should be individualized depending on the clinical and anatomical characteristics and comorbidities of each patient.

## Funding

J.B. is supported by a doctoral scholarship from 10.13039/501100000024Canadian Institute of Health Research (CIHR). L.T. was supported by a doctoral scholarship from 10.13039/501100000156Fonds de Recherche en Santé-Québec (FRSQ). J.R.-C. holds the Famille Jacques Larivière Chair in Structural Heart Disease. P.P. holds the Canada Research Chair in Valvular Heart Disease, and this study was funded, in part, by a research grant from (grant # FDN-143225; Ottawa, Ontario, Canada).

## Disclosure statement

No potential conflict of interest was reported by the authors.
