# Supplementary material for: Hemodynamic and Clinical Outcomes in Redo-Surgical Aortic Valve Replacement vs. Transcatheter Valve-in-Valve
Source: Struct Heart. 2022 Oct 28;6(6):100106. doi: 10.1016/j.shj.2022.100106 (PMC10242565; doi:10.1016/j.shj.2022.100106)
Supplement: Supplemental Figures 1-4 and Tables 1-9 [file mmc1.docx]

SUPPLEMENTAL MATERIALS

Hemodynamic and Clinical Outcomes in Redo Surgical Aortic Valve Replacement versus Transcatheter Valve-in-Valve

Sébastien Hecht, MSc; Anne-Sophie Zenses, PhD; Jérémy Bernard, MSc; Lionel Tastet, MSc; Nancy Côté, PhD; Leonardo de Freitas Campos Guimarães, MD; Jean-Michel Paradis, MD; Jonathan Beaudoin, MD; Kim O’Connor, MD; Mathieu Bernier, MD; Eric Dumont, MD; Dimitri Kalavrouziotis, MD; Robert Delarochellière, MD; Siamak Mohammadi, MD; Marie-Annick Clavel, DVM, PhD; Josep Rodés-Cabau, MD; Erwan Salaun, MD, PhD; Philippe Pibarot, DVM, PhD

Supplemental Figure Legends

**Supplemental Figure 1.** **Short-term procedural- or valve related issues and early performance of the valve according to the type of reintervention (VARC-3)**

Caption: Incidence of device success, IHP, severe PPM, AR ≥ moderate, MG ≥ 20 mmHg and hemodynamic futility following Redo-SAVR and ViV-TAVR. There was a significant higher rate of device success and IHP with Redo-SAVR (vs. ViV-TAVR) (p<0.001) but also a significant lower incidence of severe PPM, AR ≥ moderate, MG ≥ 20 mmHg and hemodynamic futility. AR, aortic regurgitation; IHP, intended hemodynamic performance; MG, mean transvalvular gradient; PPM, patient-prosthesis mismatch; Redo-SAVR, redo-surgical aortic valve replacement; ViV-TAVR, t**ranscatheter valve-in-valve.**

**Supplemental Figure 2.** **Post-procedural All-Cause Death According to the Type of Reintervention and Surgical Concomitant Procedures**

Caption: Post-procedural all-cause death according to the type of reintervention and surgical concomitant gestures (ViV-TAVR [green line], Redo-SAVR* represents patients who underwent redo surgical aortic valve replacement and any other surgical concomitant gesture, except mitral valve and/or tricuspid valve [orange line]. Redo-SAVR** represents patients who underwent redo surgical aortic valve replacement and concomitant gesture on mitral valve and/or tricuspid valve [red line]. **HR, hazard ratio;** Redo-SAVR, redo-surgical aortic valve replacement; ViV-TAVR, **transcatheter valve-in-valve.** A significant difference between groups (Log rank p=0.005) has been found. There was a trend toward higher rate of death in the ViV-TAVR vs Redo-SAVR* (HR [95% CI]: 1.95 [0.88-4.33], p=0.100) and a significant higher rate of death in the Redo-SAVR** vs Redo-SAVR* (HR [95% CI]: 2.65 [1.44-4.85], p=0.002).

**Supplemental Figure 3.** **Subgroup Analysis of All-Cause Mortality (ViV-TAVR vs Redo-SAVR)**

Caption: Subgroup analysis of all-cause mortality (ViV-TAVR vs Redo-SAVR) according to baseline characteristics. AS, aortic stenosis; AR, aortic regurgitation; BP, bioprosthesis; CAD, coronary artery disease; LVEF, left ventricular ejection fraction; MG, mean transvalvular pressure gradient; MR, mitral regurgitation; PPM, prosthesis-patient mismatch; TR, tricuspid regurgitation.

Supplemental Figure 4. Proposed Algorithm of Decision

Caption: Proposed algorithm of decision. BP, bioprostheses; CAD, coronary artery disease; MR, mitral regurgitation; PPM, prosthesis-patient mismatch; SVD, structural valve degeneration; TR, tricuspid regurgitation.

Supplemental Table 1. Variables Used in the Propensity Score Calculation

| Variables |
| --- |
| - Age - Sex - Body mass index - Chronic obstructive pulmonary disease - Renal insufficiency - Pacemaker - Coronary artery disease - EuroSCORE II - Aortic regurgitation grade - Time to failure - Bioprosthesis size (≤ 21 mm) - Pre-existing PPM |
| Legends as in Table 1 and 2. |

Supplemental Table 2. Baseline Characteristics of the Total Cohort and According to the Type of Reintervention at the Time of the Initial SAVR

|  | Total Cohort  n = 184 | Redo-SAVR  n = 104 (56.5%) | ViV-TAVR  n = 80 (43.5%) | P value |
| --- | --- | --- | --- | --- |
| Surgical BP type, n (%) |  |  |  | 0.882 |
| Stented bioprosthesis | 130 (70.6) | 73 (70.2) | 57 (71.3) |  |
| Stentless bioprosthesis | 54 (29.3) | 31 (29.8) | 23 (28.7) |  |
| Surgical BP model, n (%) |  |  |  | - |
| Mitroflow | 33 (17.9) | 16 (15.3) | 17 (21.3) |  |
| Mosaic | 37 (20.1) | 22 (21.1) | 15 (18.8) |  |
| Magna | 24 (13.0) | 15 (14.4) | 9 (11.3) |  |
| Magna Ease | 6 (3.3) | - | 6 (7.5) |  |
| Perimount | 8 (4.3) | 3 (2.8) | 5 (6.5) |  |
| Intact | 2 (1.1) | - | 2 (2.5) |  |
| Hancock | 1 (0.5) | - | 1 (1.3) |  |
| Solo | 2 (1.1) | 1 (0.9) | 1 (1.3) |  |
| Trifecta | 6 (3.3) | 5 (4.8) | 1 (1.3) |  |
| Epic Supra | 5 (2.7) | 4 (3.8) | 1 (1.3) |  |
| Freestyle | 40 (21.7) | 31 (29.8) | 9 (11.3) |  |
| Toronto | 7 (3.8) | 7 (6.7) | - |  |
| Homograft | 12 (6.5) | - | 12 (15.1) |  |
| Cryolife | 1 (0.5) | - | 1 (1.3) |  |
| Legends: BP, bioprosthesis; PPM, prosthesis-patient mismatch; ViV-TAVR, valve-in-valve transcatheter aortic valve replacement; Redo-SAVR, redo surgical aortic valve replacement. | | | | |

Supplemental Table 3. Procedural Data According to Type of Reintervention

|  | Redo-SAVR  n = 104 (56.5%) | ViV-TAVR  n = 80 (43.5%) | P value |
| --- | --- | --- | --- |
| Concomitant coronary revascularization †, n (%) | 34 (32.7) | 26 (32.5) | 0.980 |
| Aortic annulus enlargement / Aortic root replacement, n (%) | 22 (21.1) | - | - |
| Ascending aorta replacement, n (%) | 25 (24.0) | - | - |
| Mitral valve repair, n (%) | 2 (1.9) | - | - |
| Mitral valve replacement, n (%) | 20 (19.2) | - | - |
| Tricuspid valve repair, n (%) | 6 (5.8) | - | - |
| Surgical bioprosthesis size (≤ 21 mm), n (%) | 34 (42.0) | - | - |
| Surgical bioprosthesis type, n (%) |  |  | - |
| Mechanical | 21 (20.2) | - |  |
| Stented bioprosthesis | 72 (69.2) | - |  |
| Stentless bioprosthesis | 8 (7.6) | - |  |
| Sutureless bioprosthesis | 1 (1) | - |  |
| Homograft | 2 (1.9) | - |  |
| Transcatheter access site, n (%) |  |  | - |
| Transfemoral | - | 47 (58.8) |  |
| Nontransfemoral | - | 33 (41.2) |  |
| Transcarotid | - | 6 (7.5) |  |
| Transapical | - | 24 (30.0) |  |
| Transaortic | - | 3 (3.7) |  |
| Bioprosthetic valve fracture | - | 0 (0.0) | - |
| THV model, n (%) |  |  | - |
| SAPIEN | - | 39 (48.7) |  |
| SAPIEN XT | - | 8 (10.0) |  |
| SAPIEN 3 | - | 3 (3.7) |  |
| Corevalve | - | 10 (8.0) |  |
| Evolut R | - | 18 (27.0) |  |
| Portico | - | 2 (2.5) |  |
| THV size (≤23), n (%) | - | 55 (68.9) | - |
| Legends: † Concomitant coronary revascularization refers to coronary artery bypass graft performed during the redo surgical aortic valve replacement or to percutaneous coronary intervention performed during the month before the valve-in-valve transcatheter aortic valve replacement. THV, transcatheter heart valve. Other abbreviations as in Supplemental Table 2. | | | |

Supplemental Table 4. Procedural and In-Hospital Outcomes According to Type of Reintervention

|  | Redo-SAVR  n = 104 (56.5%) | ViV-TAVR  n = 80 (43.5%) | P value |
| --- | --- | --- | --- |
| Procedural and in-hospital outcomes |  |  |  |
| THV malposition or embolization, n (%) | - | 2 (2.5) | - |
| More than one THV, n (%) | - | 8 (10.0) | - |
| Myocardial infarction or coronary obstruction, n (%) | 0 (0) | 3 (3.7) | 0.082 |
| Stroke/TIA, n (%) | 7 (6.8) | 2 (2.5) | 0.303 |
| Dialysis, n (%) | 5 (4.8) | 0 (0.0) | 0.158 |
| New onset of atrial fibrillation, n (%) | 29 (28.2) | 5 (6.8) | <0.001 |
| Need of pacemaker, n (%) | 11 (10.7) | 3 (3.8) | 0.097 |
| Hospital stays, days | 9.7 ± 5.5 | 6.8 ± 4.9 | <0.001 |
| Non-transfemoral | - | 8.42 ± 5.47 | - |
| Transfemoral | - | 5.65 ± 4.08 | - |
| Redo-SAVR | 9.06 ± 4.81 | - | - |
| Redo-SAVR and concomitant surgical act | 9.97 ± 5.83 | - | - |
| Short and midterm mortality |  |  |  |
| 30-day all-cause mortality | 9 (8.7) | 2 (2.5) | 0.081 |
| 1-year all-cause mortality | 13 (12.5) | 6 (7.5) | 0.269 |
| Legends: TIA, transient ischemic attack. Other abbreviations as in Supplemental Table 2 and 3. | | | |

Supplemental Table 5. Factors Associated with Device Success, Intended Hemodynamic Valve Performance and Severe Prosthesis Patient Mismatch in the ViV-TAVR Group

|  | Univariate  OR [95% CI] | P value | Multivariate  OR [95% CI] | P value |
| --- | --- | --- | --- | --- |
| Factors associated with device success at 30 days |  |  |  |  |
| History of atrial fibrillation | 2.65 [0.92 – 7.63] | 0.070 | 2.53 [0.70 – 9.13] | 0.156 |
| EuroSCORE II | 1.07 [1.01 – 1.14] | 0.041 | 1.06 [0.99 – 1.15] | 0.100 |
| BP mode of failure (AS or mixed vs AR) | 0.29 [0.11 – 0.76] | 0.012 | 0.28 [0.87 – 0.90] | 0.032 |
| Factors associated with intended hemodynamic valve performance |  |  |  |  |
| Age | 0.99 [0.95 – 1.03] | 0.583 | 0.99 [0.93 – 1.07] | 0.930 |
| Female sex | 1.75 [0.68 – 4.52] | 0.245 | 1.61 [0.52 – 5.04] | 0.409 |
| Hypertension | 0.33 [0.10 – 1.14] | 0.080 | 0.52 [0.10 – 2.56] | 0.420 |
| Pre-existing PPM ≥ moderate | 0.37 [0.12 – 1.06] | 0.063 | 0.36 [0.12 – 1.13] | 0.079 |
| BP mode of failure (AS or mixed vs AR) | 0.36 [0.14 – 0.92] | 0.036 | 0.28 [0.09 – 0.87] | 0.028 |
| Factors associated with severe prosthesis-patient mismatch |  |  |  |  |
| Age | 1.06 [1.01 – 1.11] | 0.018 | 1.02 [0.95 – 1.10] | 0.558 |
| Female sex | 0.62 [0.24 – 1.59] | 0.320 | 0.63 [0.19 – 2.14] | 0.463 |
| Clinical Pacemaker | 4.68 [1.23 – 17.84] | 0.024 | 4.46 [0.76 – 26.09] | 0.097 |
| Pre-existing PPM ≥ moderate | 9.78 [1.28 – 19.53] | 0.001 | 7.58 [1.85 – 31.07] | 0.005 |
| BP mode of failure (AS or mixed vs AR) | 2.03 [0.79 – 5.24] | 0.142 | 2.02 [0.56 – 7.20] | 0.280 |
| Legends: AR, aortic regurgitation; AS, aortic stenosis. Other abbreviations as in Supplemental Table 2 to 4. | | | | |

Supplemental Table 6. Factors Associated with Device Success, Intended Hemodynamic Valve Performance and Severe Prosthesis Patient Mismatch in the Redo-SAVR Group

|  | Univariate  OR [95% CI] | P value | Multivariate  OR [95% CI] | P value |
| --- | --- | --- | --- | --- |
| Factors associated with device success at 30 days |  |  |  |  |
| Age | 1.00 [0.95 – 1.06] | 0.841 | 1.01 [0.94 – 1.07] | 0.936 |
| Female sex | 0.53 [0.24 – 1.21] | 0.131 | 0.57 [1.35 – 0.48] | 0.569 |
| EuroSCORE II | 0.98 [0.96 – 1.01] | 0.293 | 0.97 [0.94 – 1.01] | 0.084 |
| Post-Procedural LVEF ≤ 50% | 2.97 [0.99 – 8.85] | 0.051 | 2.95 [0.84 – 10.39] | 0.093 |
| Post-Procedural Severe PPM | 0.63 [0.26 – 1.51] | 0.299 | 0.53 [0.20 – 1.42] | 0.209 |
| BP mode of failure (AS or mixed vs AR) | 0.49 [0.21 – 1.15] | 0.102 | 0.60 [0.21 – 1.71] | 0.342 |
| Factors associated with intended hemodynamic valve performance |  |  |  |  |
| Age | 1.03 [0.97 – 1.09] | 0.295 | 1.02 [0.96 – 1.09] | 0.488 |
| Female sex | 0.40 [0.16 – 0.98] | 0.046 | 0.40 [0.15 – 1.07] | 0.068 |
| Body mass index, kg/m² | 0.92 [0.84 – 1.01] | 0.060 | 0.95 [0.86 – 1.05] | 0.295 |
| EuroSCORE II | 1.03 [0.99 – 1.07] | 0.169 | 1.02 [0.98 – 1.07] | 0.321 |
| BP mode of failure (AS or mixed vs AR) | 0.55 [0.21 – 1.43] | 0.223 | 0.92 [0.32 – 2.68] | 0.878 |
| Factors associated with severe prosthesis-patient mismatch |  |  |  |  |
| Age | 0.98 [0.92 – 1.04] | 0.537 | 0.97 [0.92 – 1.04] | 0.408 |
| Female sex | 2.56 [1.04 – 6.32] | 0.041 | 1.83 [0.46 – 7.30] | 0.392 |
| True Prosthesis ID < 21 mm (median) | 2.50 [1.03 – 6.03] | 0.042 | 0.51 [0.12 – 2.27] | 0.382 |
| Pre-existing PPM ≥ moderate | 1.91 [0.68 – 5.33] | 0.216 | 1.33 [0.42 – 4.20] | 0.626 |
| BP mode of failure (AS or mixed vs AR) | 0.82 [0.33 – 2.02] | 0.669 | 0.53 [0.19 – 1.48] | 0.228 |
| Legends: Abbreviations as in Supplemental Table 2 to 5. | | | | |

Supplemental Table 7. Univariate Analysis of Factors Associated with All-Cause Mortality

|  | **Univariable analysis** | |
| --- | --- | --- |
|  | Hazard Ratios [95% CI] | P value |
| ViV-TAVR vs Redo-SAVR | 2.20 [1.28 – 3.79] | 0.004 |
| **Clinical baseline variables** |  |  |
| Age, years | 1.07 [1.03 – 1.10] | <0.001 |
| Female sex | 2.23 [1.33 – 3.72] | 0.002 |
| Body mass index, kg/m² | 1.04 [0.99 – 1.09] | 0.070 |
| Hypertension | 2.12 [0.95 – 4.73] | 0.073 |
| Diabetes | 2.03 [1.22 – 3.38] | 0.006 |
| Previous myocardial infarction | 0.60 [0.34 – 1.06] | 0.08 |
| COPD | 2.69 [1.55 – 4.67] | <0.001 |
| Pacemaker | 1.22 [0.49 – 3.06] | 0.672 |
| History of CABG | 2.01 [1.21 – 3.35] | 0.007 |
| Coronary artery disease | 1.87 [1.10 – 3.19] | 0.022 |
| History of atrial fibrillation | 1.70 [1.01 – 2.86] | 0.047 |
| Cerebrovascular disease | 1.69 [0.73 – 3.93] | 0.224 |
| Renal Insufficiency | 2.79 [1.36 – 5.70] | 0.005 |
| EuroSCORE II | 1.04 [1.02 – 1.05] | <0.001 |
| **Pre-Procedural echocardiographic variables** |  |  |
| BP mode of failure (AS or mixed vs AR) | 1.73 [0.98 – 3.04] | 0.058 |
| LVEF < 50% | 1.78 [1.01 – 3.14] | 0.046 |
| MR or TR ≥ moderate | 1.65 [0.94 – 2.94] | 0.083 |
| Stroke volume index ≤ 35 ml/m^2^ | 2.00 [1.08 – 3.73] | 0.028 |
| **Post-Procedural echocardiographic variables** |  |  |
| MG ≥ 20 mmHg | 1.70 [0.99 – 2.93] | 0.054 |
| Aortic regurgitation ≥ moderate | 4.69 [1.43 – 15.37] | 0.011 |
| MR or TR ≥ moderate | 2.73 [1.55 – 4.80] | 0.001 |
| PPM ≥ moderate | 1.48 [0.72 – 3.04] | 0.290 |
| Severe PPM | 1.08 [0.63 – 1.88] | 0.773 |
| Hemodynamic futility | 0.75 [0.43 – 1.31] | 0.309 |
| Device success | 0.75 [0.43 – 1.31] | 0.036 |
| IHP | 0.63 [0.37 – 1.09] | 0.098 |
| LVEF ≤ 50% | 1.11 [0.61 – 2.02] | 0.721 |
| **Legends:** AVR, aortic valve replacement; CABG, coronary artery bypass grafting; COPD, chronic obstructive pulmonary disease; IHP, intended hemodynamic performance; LVEF, left ventricular ejection fraction; MG, mean transvalvular pressure gradient; MR, mitral regurgitation; TR, tricuspid regurgitation. Other abbreviations as in Supplemental Table 2 to 6. | | |

Supplemental Table 8. Multivariate Analyses of Factors Associated with All-Cause Mortality

|  | **Multivariable analysis** | |
| --- | --- | --- |
|  | Hazard Ratios [95% CI] | P value |
| **MODEL #1** |  |  |
| ViV-TAVR vs Redo-SAVR | 2.06 [1.10-3.88] | 0.025 |
| Age | 1.02 [0.98-1.06] | 0.317 |
| Sex | 2.61 [1.48-4.62] | <0.001 |
| COPD | 2.49 [1.37-4.53] | 0.003 |
| Renal insufficiency | 2.97 [1.35-6.52] | 0.007 |
| Pre-Procedural EuroSCORE II | 1.04 [1.02-1.06] | <0.001 |
| **MODEL #2** |  |  |
| ViV-TAVR vs Redo-SAVR | 2.54 [1.32-4.87] | 0.005 |
| Pre-Procedural EuroSCORE II | 1.05 [1.03-1.08] | <0.001 |
| BP mode of failure (AS or mixed vs AR) | 1.60 [0.74-3.43] | 0.231 |
| Pre-Procedural LVEF <50% | 0.95 [0.45-1.97] | 0.885 |
| Pre-Procedural Stroke volume index ≤ 35 ml/m^2^ | 2.29 [1.13-4.61] | 0.021 |
| Pre-Procedural MR and/or TR ≥ moderate | 1.51 [0.79-2.85] | 0.209 |
| **MODEL #3** |  |  |
| ViV-TAVR vs Redo-SAVR | 3.52 [1.73-7.13] | <0.001 |
| Pre-Procedural EuroSCORE II | 1.05 [1.03-1.08] | <0.001 |
| Post-Procedural Stroke volume index ≤ 35 ml/m^2^ | 1.40 [0.73-2.66] | 0.309 |
| Post-Procedural MR and/or TR ≥ moderate | 2.68 [1.41-5.10] | 0.003 |
| IHP | 0.67 [0.34-1.35] | 0.266 |
| **MODEL #4** |  |  |
| ViV-TAVR vs Redo-SAVR | 3.48 [1.71-7.06] | <0.001 |
| Pre-Procedural EuroSCORE II | 1.06 [1.03-1.08] | <0.001 |
| Post-Procedural Stroke volume index ≤ 35 ml/m^2^ | 1.45 [0.76-2.77] | 0.257 |
| Post-Procedural MR and/or TR ≥ moderate | 2.69 [1.41-5.10] | 0.002 |
| Device success | 0.60 [0.30-1.19] | 0.149 |
| **Legends:** Abbreviations as in Supplemental Table 2 to 7. | | |

|  | **Univariable analysis** | |
| --- | --- | --- |
|  | OR [95% CI] | p Value |
| ViV-TAVR vs Redo-SAVR | 0.97 [0.53 – 1.75] | 0.917 |
| **Clinical baseline variables** |  |  |
| Female sex | 0.59 [0.15 – 2.26] | 0.443 |
| Age, years | 0.90 [0.85 – 0.96] | <0.001 |
| Body mass index, kg/m² | 1.06 [0.96 – 1.18] | 0.256 |
| Hypertension | 1.06 [0.22 – 5.07] | 0.945 |
| Diabetes | 2.02 [0.62 – 6.53] | 0.242 |
| COPD | 0.84 [0.18 – 4.03] | 0.830 |
| History of CABG | 0.13 [0.02 – 1.00] | 0.050 |
| Coronary artery disease | 0.56 [0.17 – 1.85] | 0.346 |
| History of atrial fibrillation | 0.53 [0.11 – 2.51] | 0.426 |
| Cerebrovascular disease | 0.85 [0.18 – 4.11] | 0.840 |
| EuroSCORE II | 0.89 [0.77 – 1.02] | 0.089 |
| **Pre-Procedural echocardiographic variables** |  |  |
| BP mode of failure (AS or mixed vs AR) | 1.69 [0.44 – 6.48] | 0.443 |
| LVEF < 50% | 0.71 [0.15 – 3.41] | 0.665 |
| MR or TR ≥ moderate | 0.28 [0.34 – 2.28] | 0.243 |
| Stroke volume index ≤ 35 ml/m^2^ | 1.06 [0.21 – 5.26] | 0.943 |
| **Post-Procedural echocardiographic variables** |  |  |
| Hemodynamic futility | 1.10 [0.31 – 3.91] | 0.885 |
| MG ≥ 20 mmHg | 0.92 [0.28 – 3.02] | 0.888 |
| Severe PPM | 1.03 [0.32 – 3.32] | 0.965 |
| LVEF ≤ 50% | 1.60 [0.46 – 5.61] | 0.461 |
| **Legends:** Abbreviations as in Supplemental Table 2 to 7. | | |

Supplemental Table 9. Variables Associated with Aortic Valve Reintervention Following Redo-SAVR or ViV-TAVR

Supplemental Figure 1. Short-term procedural- or valve- related issues and early performance of the valve according to the type of reintervention (VARC-3)


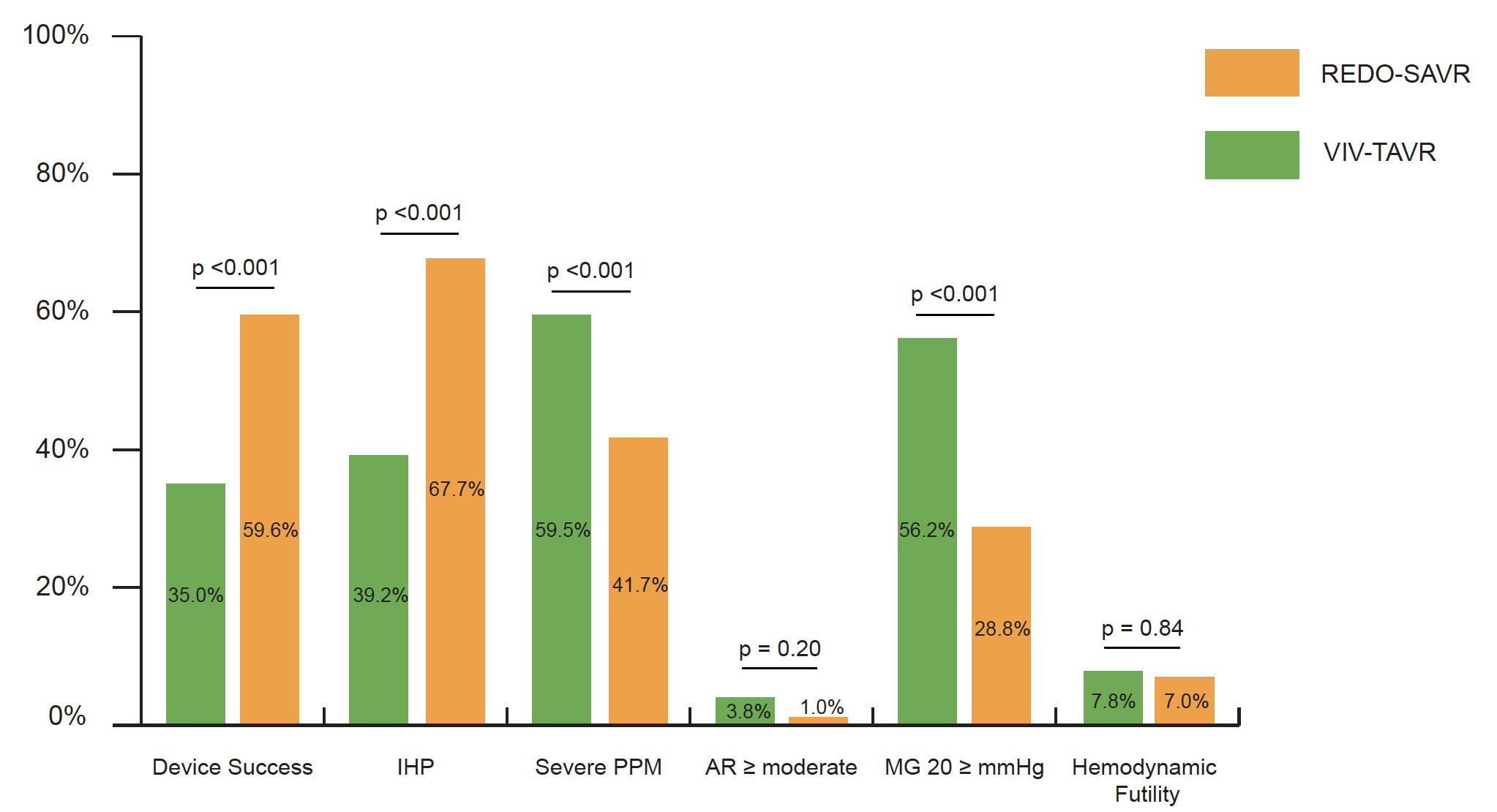


**Legends:** AR, aortic regurgitation IHP, intended hemodynamic performance; MG, mean transvalvular gradient; PPM, patient-prosthesis mismatch; Redo-SAVR, redo-surgical aortic valve replacement; ViV-TAVR, t**ranscatheter valve-in-valve.**

Supplemental Figure 2. Post-procedural All-Cause Death According to the Type of Reintervention and Surgical Concomitant Procedures


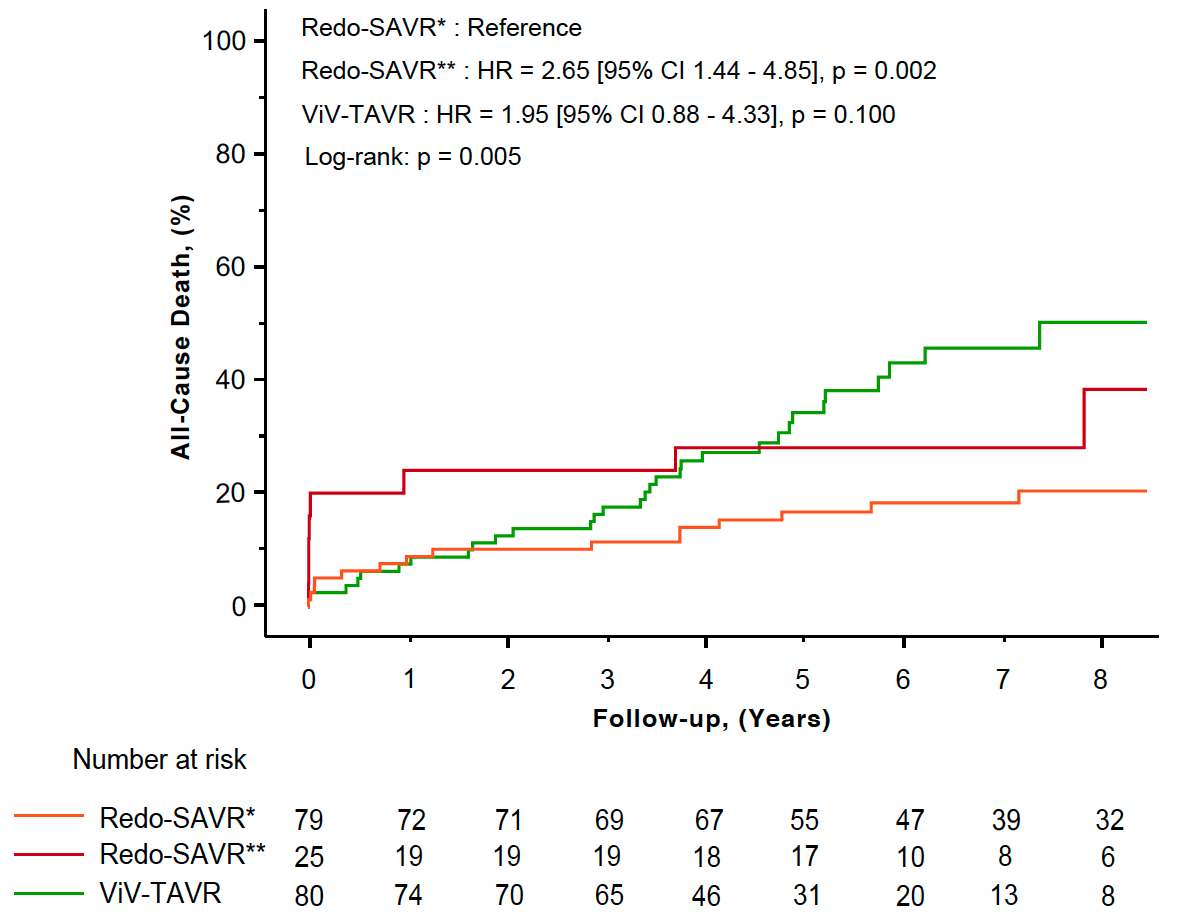


**Legends:** Redo-SAVR, redo-surgical aortic valve replacement; ViV-TAVR, t**ranscatheter valve-in-valve.**

***: Redo-SAVR** and any other surgical concomitant gesture, except mitral valve and/or tricuspid valve

****: Redo-SAVR with** concomitant gesture on mitral valve and/or tricuspid valve

Supplemental Figure 3. Subgroup Analysis of All-Cause Mortality (ViV-TAVR vs Redo-SAVR)


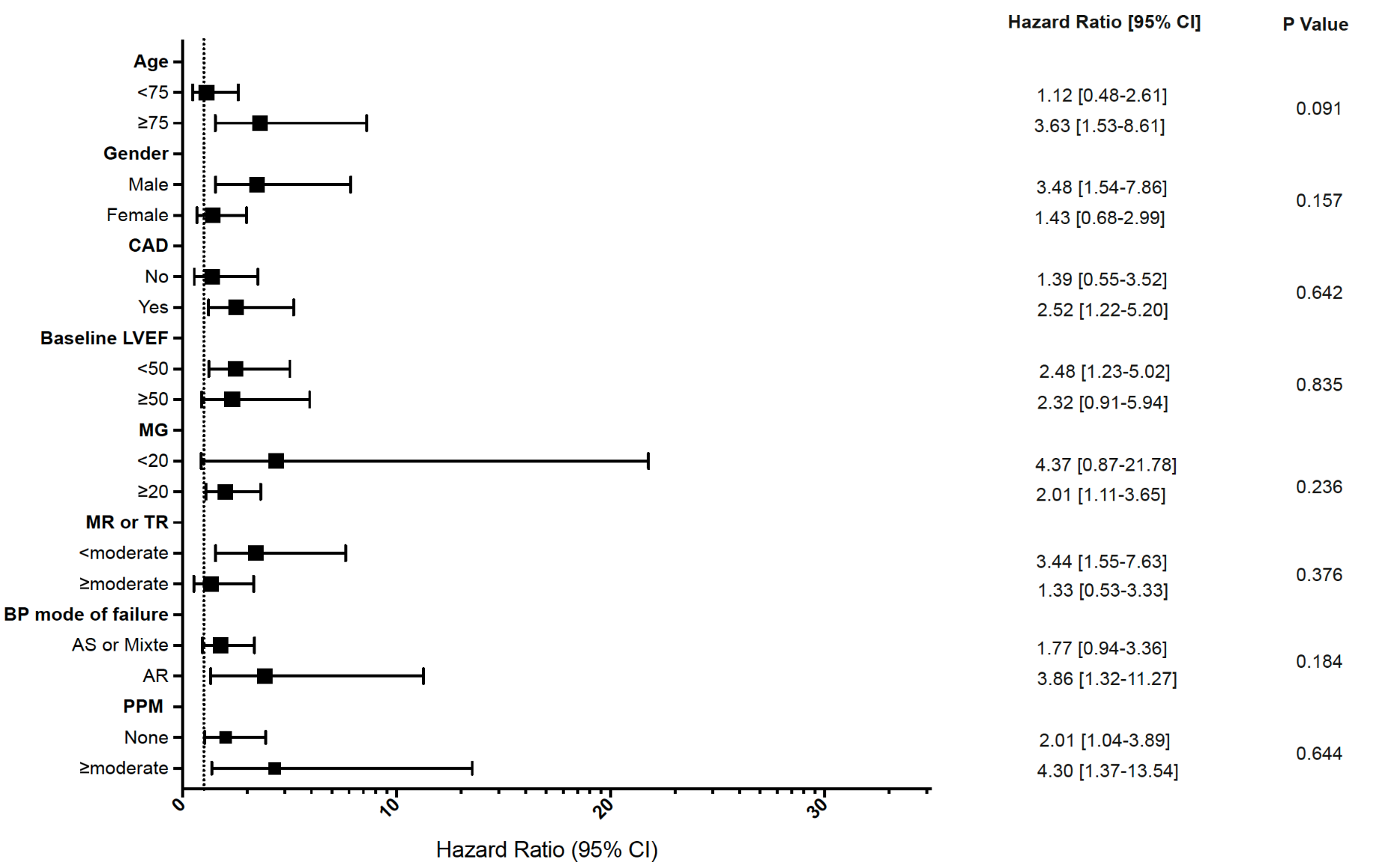


Legends: The hazard ratio is for the mortality risk in TAVR-ViV vs. Redo-SAVR. AR, aortic regurgitation; AS, aortic stenosis; BP, bioprosthesis; CAD, coronary artery disease; LVEF, left ventricular ejection fraction; MG, mean transvalvular pressure gradient; MR, mitral regurgitation; PPM, pre-existing prosthesis-patient mismatch (PPM of the failed surgical bioprosthesis); TR, tricuspid regurgitation.

Supplemental Figure 4. Proposed Algorithm of Decision


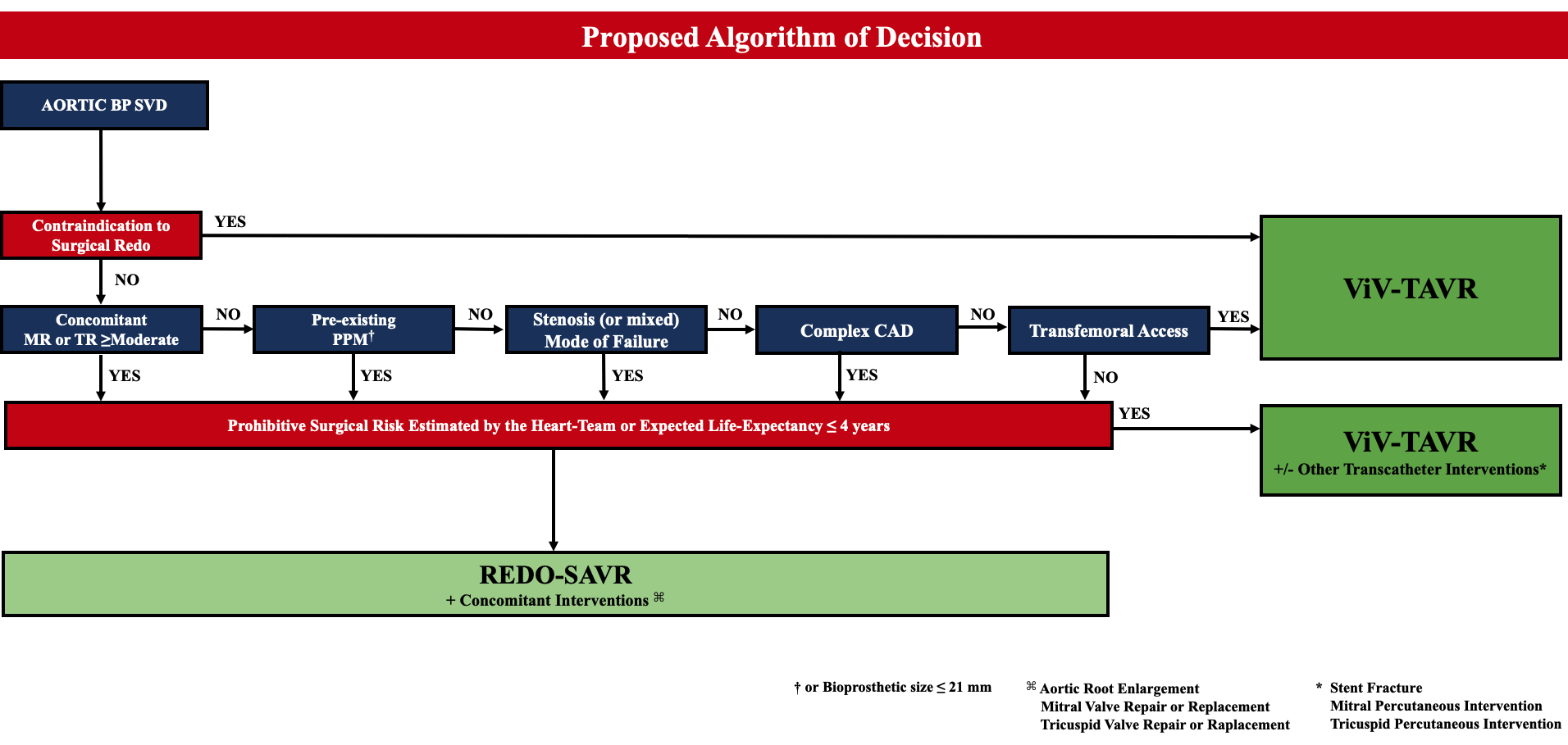


Legends: BP, bioprostheses; SVD, structural valve degeneration; other abbreviations as in Supplemental Figure 1 to 3.
